# 3-dimensional bioprinting for tissue engineering applications

**DOI:** 10.1186/s40824-016-0058-2

**Published:** 2016-04-25

**Authors:** Bon Kang Gu, Dong Jin Choi, Sang Jun Park, Min Sup Kim, Chang Mo Kang, Chun-Ho Kim

**Affiliations:** Laboratory of Tissue Engineering, Korea Institute of Radiological and Medical Sciences, 215-4, Gongneung, Nowon, Seoul, 139-240 Korea

**Keywords:** 3D bioprinting, Additive manufacturing, Tissue engineering, 3D scaffold

## Abstract

The 3-dimensional (3D) printing technologies, referred to as additive manufacturing (AM) or rapid prototyping (RP), have acquired reputation over the past few years for art, architectural modeling, lightweight machines, and tissue engineering applications. Among these applications, tissue engineering field using 3D printing has attracted the attention from many researchers. 3D bioprinting has an advantage in the manufacture of a scaffold for tissue engineering applications, because of rapid-fabrication, high-precision, and customized-production, etc. In this review, we will introduce the principles and the current state of the 3D bioprinting methods. Focusing on some of studies that are being current application for biomedical and tissue engineering fields using printed 3D scaffolds.

## Background

**Fig. 1:**
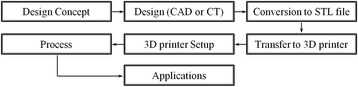
The 3D printing process. (step-by-step)

The conception of 3-dimensional (3D) printing technologies was first introduced in 1986 by Charles W. Hull [1]. It referred to as additive manufacturing (AM) or rapid prototyping (RP) has acquired reputation over the past few decades [2–4]. 3D printing is one of the additive manufacturing processes [5, 6]. 3D printing is a proper name to describe the technologies that create 3D structures by adding layer-by-layer of material, whether the material is ceramic, metal, plastic, and polymers (synthetic or natural polymers) [7]. The steps involve in product development using 3D printing are shown in Fig. 1. The 3D printing technologies are commonly used by the computer, 3D modeling software (computer-aided design (CAD) or computer tomography (CT) scan images), machine equipment and layering materials [8]. After CAD sketch, 3D printing equipment reads out data from the CAD file and then 3D structure is produced [9].

Typically, ASTM (F2792) standard terminology for 3D printing (additive manufacturing) technologies consists of several parts such as vat photopolymerization, material jetting, material extrusion, powder bed fusion, binder jetting, sheet lamination, and directed energy deposition as shown in Table 1 [10]. Briefly, the 3D printing of vat photopolymerization method has a container with a photopolymer resin and which is then hardened with ultraviolet (UV) light or another similar power source. The most commonly used technology in this process is the Stereolithography Apparatus (SLA) and the Digital Light Processing (DLP) [11, 12]. This method uses a vat of liquid photopolymer resin and UV laser to create the 3D structures one at a time. The material is fed through a nozzle with a small diameter in material injection process, and the operating behavior is analogous to the typical inkjet printer. It is produced the 3D structure to the layer-by-layer, and then cured by UV light [13]. The binder jetting process uses a liquid binding agent and a powder-based material. The print-head selectively drops the liquid binding agent into the powder materials for 3D structure. In addition, this process can print a variety of materials such as metals, ceramics, and polymers [14, 15]. The most commonly used technology in the material extrusion process is the Fused Deposition Modeling (FDM) [16, 17]. The representative method of FDM is the Fused Filament Fabrication (FFF). The FFF technology is the most common and simplest 3D printing. It uses thermoplastic filament as the printing material. The filament is melted in the head of the 3D printer through heating and then creates 3D structures by adding layer-by-layer. In the powder bed fusion process, generally used technology is the Selective Laser Sintering (SLS) [18, 19]. During the SLS process, small particles (powder) of polymer, glass, or ceramic are fused together by heat from a high power laser to form a 3D structure. The sheet lamination technology is to include different materials in the sheet by using external force. Sheets may be used the metal, plastic, polymer, etc. [20, 21]. During the sheet lamination process, the sheet is laminated together using heat and pressure, and then cut into the desired shape with a laser or blade. Finally, the directed energy deposition process is mostly used in the high-tech metal industries and in rapid manufacturing applications [22, 23]. This printing apparatus consists of a multi-axis robotic arm with a nozzle, an energy source (laser, electron, or plasma), and a substrate for deposits melted material. For creating a 3D structure, the melted materials by the energy source are deposited on the substrate through nozzles, and then harden.

**Table 1:** ASTM standard terminology for additive manufacturing technologies

Additive Manufacturing (ASM F2792)	
Vat photopolymerization	• Stereolithography (SLA)
	• Digital light processing (DLP)
Material jetting	• Multi-jet modeling (MJM)
Material extrusion	• Fused deposition modeling (FDM)
Powder bed fusion	• Electron beam melting (EBM)
	• Selective laser sintering (SLS)
	• Selective heat sintering (SHS)
	• Direct metal laser sintering (DMLS)
Binder jetting	• Powder bed and inkjet 3D printing (PBIH)
	• Plaster-based 3D printing (DMLS)
Sheet lamination	• Laminated object manufacturing (LOM)
	• Ultrasonic consolidation (UC)
Directed energy deposition	• Laser metal deposition (LMD)

Recently, the aim of tissue engineering is regeneration, restoration, or replacement of defective or injured functional living organs and tissues [24–26]. In order to achieve this aim, biomedical scaffolds made of natural or synthetic polymers have been commonly used in biomedical and tissue engineering applications [27, 28]. The major focus of these scaffolds is to replace or regenerate the native tissues functionally and structurally. In general, the scaffolds for use as tissues and organs have a several mandatory functions: it should provide internal pathways for the cell attachment and migration, it must transfer various growth factors and waste products, and it should keep its shape while the cells are growing, and have adequate mechanical properties. [29]. To achieve these functions, biomedical scaffolds for tissue engineering require a highly porous 3D structure that allows cell affinity such as proliferation, migration, attachment, and differentiation, even enables nutrients and oxygen transport [30, 31]. Therefore, 3D bioprinting technology is one of the most appropriate methods for producing a 3D structure for use as biomedical scaffolds, tissues, and organs. The 3d bioprinting is the technique for controlling a cell pattern to be retained functionality and viability of the cells within the printed 3D structure. In tissue engineering, development of the appropriate scaffold using a 3D printing has already been studied by many researchers [32, 33]. Advances introduced by 3D bioprinting have importantly enhanced the ability to control pore size distribution, pore volume, and pore interconnectivity of scaffolds. Furthermore, 3D bioprinting accredit to important advances in tissue engineering field by the study of biomaterials or bio-ink. Development of biomaterials in 3D bioprinting is an important prerequisite to a direct effect on cell growth. Some 3D printing processes to contain living cells and bioactive molecules in biomaterials (hydrogels) made successfully 3D structures at room temperature without any significant effect on the cell viability. For applications using 3D bioprinting technologies in tissue engineering, researchers should be considered the biomaterials (bio-ink) as well as the 3D structure (design).

Among additive manufacturing technologies, several methods such as SLA [34, 35], FFF [36, 37], SLS [38, 39] and inkjet 3D printing [40, 41], etc. have been applied in tissue engineering field. These methods have been used in various sectors as architectural modeling, art, and lightweight machines and also 3D structures from biomaterials is used for tissue engineering and regenerative medicine. 3D bioprinting is to produce a 3D structure of the desired shape by combining the living cells and biomaterials. Researchers are developing various methods to fabricate 3D unique structure with biological and mechanical properties suitable for regeneration of native tissue. In this review, we describe the four different type of 3D bioprinting technology for fabrication of 3D structure and its application in tissue engineering and regenerative medicine fields.

## Review

### 3D bioprinting for tissue engineering application

3D Bioprinting form biomaterials are an emerging technology which aims to develop new organs and tissues. This technology is currently in research phase, and many researchers have conducted a study. 3D bioprinting is a process for controlling the cell proliferation, attachment, and migration within 3D structures [42, 43]. Therefore, various 3D bioprinting methods are used for a variety of tissue engineering applications. Herein, we will introduce the four types of 3D bioprinting methods that are most commonly used such as SLA and DLP in vat photopolymerization, FFF in material extrusion, SLS in powder bed fusion, and inkjet 3D printing in binder jetting methods. Table 2 shows advantages and disadvantages of various 3D bioprinting methods for tissue engineering applications.

**Table 2:** Advantages and disadvantages of various 3D bioprinting methods for tissue engineering applications

Methods	Advantages	Disadvantages	Materials	Ref.
SLA, DLP	• Manufactured simple and complex	• Expensive equipment and materials	PEG, PCL, PEG-*co*-PDP, PEGDA.	[45–48]
	• Fast and good resolution	• Only photopolymers		
	• No need for support materials	• Cytotoxicity of uncured photoinitiator		
FFF	• Easy to use	• Materials limited to thermoplastics	PCL/PLGA/*β*-TCP, PCL/PLGA	[50, 51]
	• Good mechanical properties	• Filament required		
	• Solvent not required	• Cannot used with cells		
SLS	• No need for support materials	• Rough surface	PCL/HA, PCL, HA/PEEK, Titanium.	[59–62]
	• Various of biomaterials	• Expensive and cumberstone equipment		
Inkjet	• Cells and hydrogel printed	• Limited biomaterials suite	Collagen/PDL, Fibrin, Gelatin.	[63–65, 68]
	• Incorporation of drug and biomolecules	• Low resolution		
		• Low mechanical properties		

### Vat photopolymerization method

**Fig. 2:**
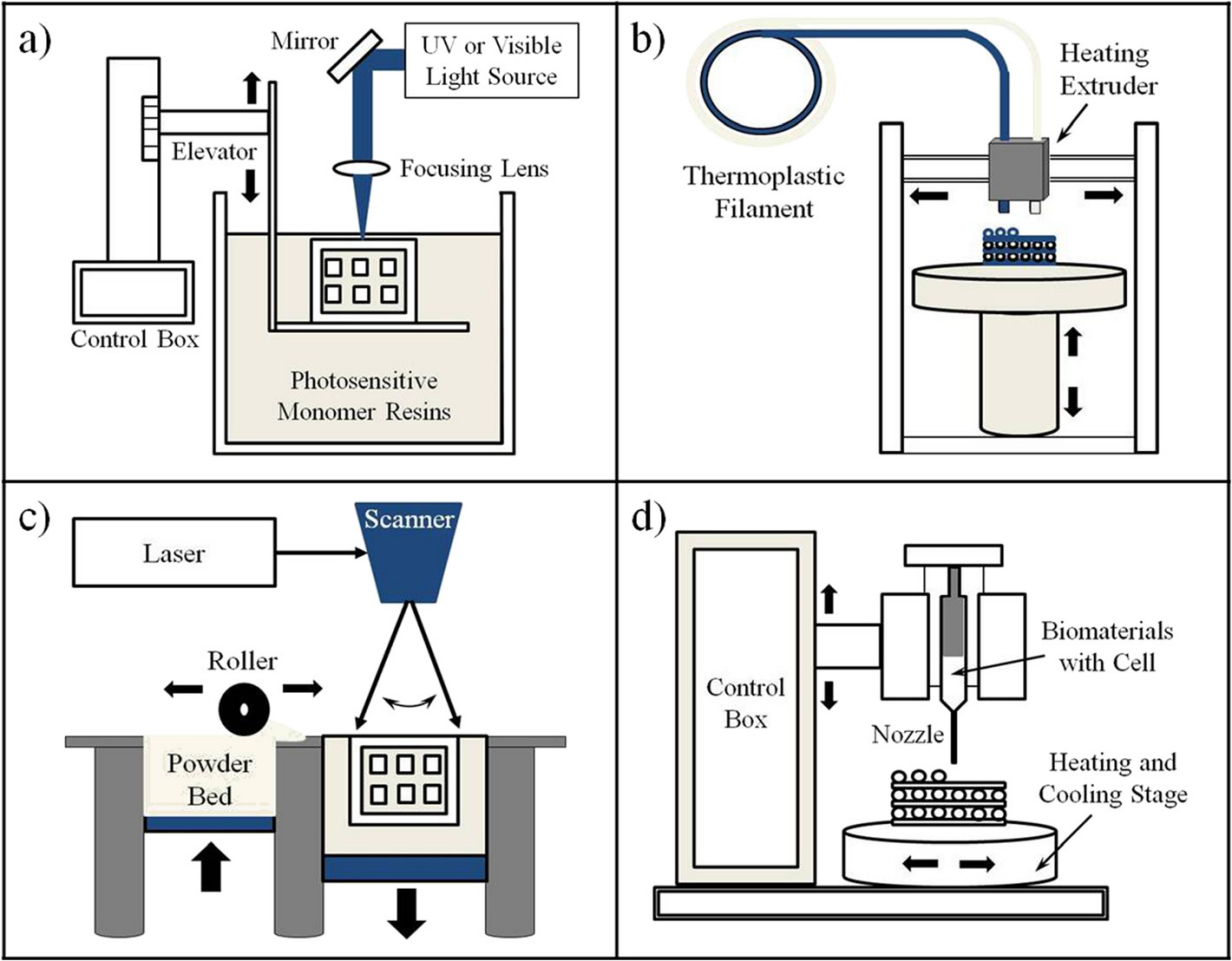
Schematics of various 3D bioprinting for tissue engineering applications; **a** Vat photopolymerization, **b** Fused filament fabrication, **c** Selective laser sintering, **d** Inkjet 3D printing

SLA method using the UV light is one of the various methods used to create the 3D structures. This method has been the oldest and still widely used. This process has obtained the patent in 1986 by Charles Hull [1]. Also, DLP is similar to the SLA method. The main difference of the DLP is to use a visible light source, such as a liquid crystal display panel and an arc lamp. SLA and DLP are based on the vat photopolymerization principle of photosensitive monomer resins when exposed to UV light or another similar power source. Photopolymerization is driven by a chemical reaction that produces free radicals when exposed to certain wavelengths of light. Photons from the light source dissociate the photoinitiator to a high energy radical state. The radical induces the polymerization of the macromer or monomer solution. However, the problem with this photopolymerization process is that the created free radicals can have damage to the cell membrane, protein, and nucleic acids. Therefore, it is important to find a cytocompatible photo-initiator for the SLA 3D printing method. A typical schematic of vat photopolymerization method is shown in Fig. 2(a). To obtain the 3D scaffold for tissue engineering application, many researchers reported the SLA product with various biomaterials. Neiman et al. have fabricated composite 3D structures with photopolymerizable PEG based hydrogel scaffolds using SLA based process. The aim of this study was development to foster formation of 3D liver aggregates and microperfusion flow within the open channels of this structure [44]. Elomaa et al. showed that they used the _L_-alanine-derived depsipeptide to synthesize a new biodegradable, photopolymerizable poly(ethylene glycol-*co*-depsipeptide) macromer for the DLP-based fabrication of cell-laden hydrogel constructs for vascular applications [45]. In addition, they (Elomaa et al.) also reported that three-armed polycaprolactone (PCL) oligomers of various molecular weights were synthesized, end-functionalized with methacrylic anhydride and photopolymerized. PCL-based photopolymerizable and biodegradable resins were formulated and used without solvents in SLA to accurately prepare designed porous 3D scaffolds [46]. In resulting 3D scaffolds, they have explained that 3D structures with a high porosity have a great potential in the cell culture and implanting. Chan et al. introduced that cell encapsulated hydrogel with a complex 3D structure was created from photo-polymerizable poly(ethylene glycol) diacrylate (PEGDA) using modified SLA method [47]. In the result, NIH/3 T3 cells-encapsulated within 3-Dimensional structures were successful, and it was confirmed that the cells are excellent in affinity (cell viability, proliferation and spreading). Seck et al. described the synthesis of poly(ethylene glycol)/poly(_D,L_-lactide) based macromers, the resin formulation, and the photo-polymerization process by DLP that allows the generation of designed 3D crosslinked structures [48]. The fabricated porous hydrogel structure showed narrow pore size distributions, excellent pore inter-connectivity and enhanced mechanical properties. The human mesenchymal stem cells on this 3D structure exhibit a characteristic of superior cell adhesion and proliferation.

The main advantages of vat photopolymerization method in tissue engineering applications are that fabrication of simple, complex designs, fast processing, high resolution, and no need for support material. The disadvantages are that expensive equipment, expensive curing materials as photoinitiator, and cytotoxicity of uncured photoinitiator.

### Fused filament fabrication method

FFF printers in material extrusion method use a thermoplastic filament. This filament is heated to the melting point and then extruded to prepare a 3D structure. These thermoplastic filaments are deposited through an extrusion nozzle during printing. The nozzle melts the filaments and then extrudes onto the substrate for fabricating 3D structure (FFF method). The nozzle and substrate are controlled by a computer that translates the dimensions of a structure into X, Y and Z coordinates during printing. A schematic of material extrusion method is shown in Fig. 2(b). FFF method is a thermal-heating technique for use 3D scaffolds fabrication in tissue engineering applications. Many researchers were reported using FFF method for tissue engineering. Pati et al. reported that to enhance the biological properties of extracellular matrix (ECM)-ornamented 3D printed scaffolds with cells using FFF bioprinting [49]. They developed bone graft substitutes by using 3D printed scaffolds made from a composite of polycaprolactone (PCL), poly(lactic-co-glycolic acid) (PLGA), and *β*-tricalcium phosphate and mineralized ECM laid by human nasal inferior turbinate tissue-derived mesenchymal stromal cells. Lee et al. fabricated melt-plotted/in situ plasma-treated PCL scaffolds coated with chitosan of various molecular weights in a layer-by-layer manner [50]. They evaluated the effects of the chitosan coating on various physical and cellular activities, including water wetting ability, cell proliferation, ALP activity, and calcium deposition using the osteoblast-like MG63 cell line. Hong et al. fabricated solid freeform fabrication based 3D PCL/PLGA scaffolds that provide functionalized surfaces through a simple but efficient coating of mussel adhesive proteins without any surface modification procedures [51].

The main advantages of FFF method in tissue engineering applications are that easy to use, a variety of biomaterials, good mechanical properties, and the solvent not required. The disadvantages are material restriction related to thermoplastic polymers. In addition, it cannot be printed with the cells due to the high manufacturing temperature.

### Selective laser sintering method

SLS is a technique that uses the laser as a power source to form solid 3D structures. This method uses a high power laser for powder sintering to form a scaffold. This method is produced by selective laser printing from 3D modeling software in the part on the surface of a powder bed. This process may be printed from several of materials such as ceramics, metals, and polymers. A schematic of SLS is shown in Fig. 2(c). SLS of polymer powder has been evaluated by several groups for tissue engineering application and drug delivery system [52–55]. Moreover, the SLS has been used to tissue engineering application as scaffolds from polymeric biomaterials and their composites [56–58]. Du et al. fabricated a novel protocol to produce SLS-derived bone scaffolds using the PCL microspheres and polycaprolactone/hydroxyapatite (PCL/HA) composite microspheres as the basic building materials [59]. The biocompatible evaluation of the SLS-derived scaffolds was investigated using rat MSCs and the results showed both pure PCL scaffolds and PCL/HA composite scaffolds can well support cell adhesion, proliferation, and growth. Williams et al. used SLS to process PCL to produce parts with controlled pore sizes in the range 1.75 ~ 2.5 mm and designed porosities from 63.1 % to 79 %, but met with limited success in terms of accurately achieving the required porosity levels [60]. Particle size and thermodynamic variations were found to play critical roles. Tan et al. demonstrated the ability of SLS to fabricate physically blended hydroxyapatite/poly(ether-ether-ketone) composites for tissue scaffold development and observed micropores on the scaffold surface [55]. Chen et al. showed that PCL scaffolds manufactured by SLS were surface modified by immersion coating with either gelatin or collagen for cartilage tissue engineering [61]. Ciocca et al. reported a technique to design and manufacture a customized titanium mesh for minimal bone augmentation of an atrophic maxillary arch, guided by the final position of the prosthesis and according to the implants necessary for its support [62].

The main advantages of this process for tissue engineering applications are a wide range of biomaterials that can be used. Powder bed is used as a support, therefore, no need for secondary support structures. Also, unused powders may be recycled. The disadvantage of SLS is that the detail is not as crisp and sharp when compared with other processes, such as SLA and FFF. Another disadvantage is that the SLS bioprinters tend to be large, cumbersome, and expensive.

### Inkjet 3D printing

Inkjet 3D printing method is a rapid prototyping and layered manufacturing technology for making structures described by 3D modeling data. Inkjet 3D printing is closely related to Inkjet head printing. Lately, inkjet 3D printing method has been significant developments in the use of polymeric bio-ink printing for applications in biological and tissue engineering fields. A schematic of inkjet 3D printing is shown in Fig. 2(d). Inkjet bioprinters are the most commonly used type of printer for both non-biological and biological applications. Many researchers were reported using inkjet head 3D bioprinting method for tissue engineering. Sanjana et al. reported on the use of inkjet bioprinting to create neuron adhesive patterns as islands and other pattern using PEG (cell-repulsive material) and collagen/poly-_D_-lysine mixture (cell-adhesive material) [63]. Xu et al. use the inkjet bioprinting technology for the fabrication of 3D scaffolds, based on fibrin gel [64]. Fibrin has been used as a printable hydrogel for building a 3D neural construct. Lee et al. reported the printing of a growth factor-releasing fibrin gel containing murine neural stem cells (NSCs) to construct an artificial neural tissue and then examined the effects of the growth factor-releasing fibrin gel on the survival of the murine NSCs [65]. Lorber et al. printed retinal glia cells with cell culture media and subsequently assessed the survival of these cells in culture [66]. Pati et al. have focused on bioprinting of dome-shaped adipose tissue constructs using human decellularized adipose tissue matrix bio-ink that encapsulates human adipose tissue-derived mesenchymal stem cells through biomimetic approach for evaluation of their efficacy in adipose tissue regeneration [67]. Irvine et al. reported on the development of printable gelatin as the bio-ink with cell-encapsulated. They were fabricated patterned 3D structure by using inkjet bioprinter and then confirmed excellent cell affinity [68].

The advantages of inkjet 3D bioprinting method for tissue engineering applications are that patient-customized fabrication, rapid production, low cost of production, and easy to incorporate both drug and biomolecules. In addition, it can be a printing with the cells. The disadvantages are that limitation of size and biomaterials, low resolution, and negligible mechanical properties.

### Current and future direction for 3D bioprinting

The technology for 3D bioprinting has a lot of advantages, but it still has many challenges that remain to be overcome. Heretofore, several types of research about 3D bioprinting have conducted in the lab of universities and companies. For example, Organovo’s exVive3D™ Liver bioprinted human tissue models with collagen are created using proprietary 3D bioprinting technology [69]. The resulting tissues contain accurate and reproducible 3D structure that can remain completely functional and reliable over 40 days. Also, Atala group was succeeded in scaffold production for the human kidney using 3D bioprinting technology [42]. Cornell university researchers reported that 3D printed ears similar to human ear using 3D bioprinting and collagen gels with living cells [70]. So far, as mentioned above, patient-customized 3D bioprinting was studied only in a few laboratories. However, 3D bioprinting in the future has to be the development of various models in many laboratories.

Additionally, the development of bio-inks (biomaterials) in 3D bioprinting is very important for printing tissue or organs in the future. However, 3D bioprinting processes are limited to scaffolds for cells support and simple body parts such as bone. Currently, most of the bio-inks for 3D bioprinting are limited to collagen, gelatin, fibrin, ceramics, thermoplastics or light-curable composite. As shown in Table 3, the range of available bioprinting materials is limited. To overcome these limitations, the development of new biomaterials that can be printed with the cells is necessary. The biomaterials for bioprinting should be biocompatible, easily manufactured, sufficient mechanical properties for cells support, secure 3D structure.

**Table 3:** Bio-inks for 3D bioprinting

Manufacturers	Names	Bio-inks	Applications	Ref.
Organovo Holdings	exVive3D™ Human Liver Models	Collagen	Drug screening	[69]
Cornell Univ.	-	Collagen	Ear	[70]
Wake Forest Univ.	-	Kidney cell, nephron	Kidney	[42]
Washington Univ.	-	Ceramic powder	Bone	[31]
RegenHU	BioInk™	Collagen, Fibrin	Soft tissue	[71]
RegenHU	OsteoInk™	Collagen, Calcium phosphate	Hard tissue	[72]
EnvisionTEC Gmbh	E-Shell series	Acrylonitrile butadiene styrene (ABS)	Hearing aid	[73]
EnvisionTEC Gmbh	E-Dent Series	Light-curable composite	Dental	[74]

The future of 3D bioprinting is not limited to inanimate structures. 3D printed medical implants will be able to enhance the quality of human life. 3D bioprinting is currently used for prosthetic limbs, orthodontic devices, and bone implants because it can be matched to the correct body shape of the patient. Printing of soft tissue is progress, and can be used immediately in veins and arteries printing operations. Today, medical applications of 3D bioprinting have developed a nano-medicine, pharmaceuticals, and organs such as human health fields. Finally, direct organ fabrication using 3D bioprinting technology is the ultimate goal in tissue engineering and regenerative medicine. There is a possibility of printing a complete organ that could be directly transplanted into the human body.

## Conclusion

In the recent years, a lot of 3D bioprinting method and design has been developed for tissue engineering. Especially, computer-aided 3D printing techniques have a great potential to fabricate complex 3D structures with highly porosity architecture. It can be achieved great strides in biomedical application fields, especially infusion of medical imaging techniques such as CT and MRI. However, the low resolution and using only one technology for fabricating a native tissue similar 3D structure, there is a limit. Thus, using more than two 3D printing technologies or combination of 3D printing technologies with other scaffold fabrication technologies can overcome the limitations and fabricate a multifunctional 3D structure. In the recent, only a few of the research groups have been deeply characterized though extensive in vitro and in vivo studies and results are mostly limited to a restricted number of biomaterials. Thus, development of materials (bio-ink) is one of the most important goals in 3D printing. It has enabled to directly create implantable devices such as biodegradable tissue engineering scaffolds.
